# Risk factors for irreversible unilateral loss of renal function in patients with deep endometriosis

**DOI:** 10.1038/s41598-023-38728-z

**Published:** 2023-07-24

**Authors:** María-Angeles Martínez-Zamora, Eduard Mensión, Judith Martínez-Egea, Lluis Peri, Agustín Franco, Meritxell Gracia, Cristina Ros, Mariona Rius, Francisco Carmona

**Affiliations:** 1 Department of Gynecology. Institute Clinic of Gynecology, Obstetrics and Neonatology, Hospital Clinic of Barcelona. Faculty of Medicine, Institut d´Investigacions Biomèdiques August Pi i Sunyer (IDIBAPS), University of Barcelona, Villarroel St., 170, 08036 Barcelona, Spain; 2 Urology Department, Hospital Clinic of Barcelona, Barcelona, Spain

**Keywords:** Medical research, Urology

## Abstract

Deep endometriosis (DE) can be more aggressive than other types of endometriosis, and may even lead to irreversible severe complications such as complete unilateral loss of renal function. We aimed to describe the clinical and radiologic characteristics of DE patients diagnosed with irreversible unilateral loss of renal function due to unilateral ureteral stenosis and evaluate risk factors for developing this loss. This retrospective cohort study included 436 patients who underwent laparoscopic DE surgery. We evaluated two groups of patients according to preserved (Non-Renal Loss Group; n = 421) or irreversible unilateral damaged renal function (Renal Loss Group; n = 15). Preoperative epidemiologic variables, clinical characteristics, radiologic findings and surgical treatments of all the patients were collected. The Renal Loss Group had a higher infertility rate and a higher proportion of asymptomatic patients. The following radiological variables showed statistically significant differences between the two groups: mean endometrioma diameter, the presence of intestinal DE and negative sliding sign. Multivariate analysis showed that infertility, being asymptomatic, having intestinal DE or torus uterinus/uterosacral ligament DE and a negative sliding sign significantly increased the risk of loss of renal function. Therefore, among patients with these clinical and/or radiological variables, severe urinary tract obstruction should be specifically ruled out.

## Introduction

Deep endometriosis (DE) is usually associated with severe pelvic pain, dysmenorrhea and other pain symptoms and can be more aggressive than other types of endometriosis, and may even lead to irreversible severe complications such as complete unilateral loss of renal function^1,2^. However, severe urinary tract DE is often less symptomatic or even asymptomatic, and may be diagnosed incidentally by hydronephrosis found on abdominal ultrasound or magnetic resonance imaging (MRI)^3^ or during a surgical procedure for other symptomatic DE locations. Furthermore, previous data showed that up to 30% of patients with urinary tract DE may have reduced kidney function at the time of diagnosis^4^ although the real prevalence is unknown. This late diagnosis is the result of the clinically silent form of DE among these patients^5^, being first described not many years ago^6^. Thus, when there is the suspicion of ureteral obstruction in DE patients, the diagnostic approach should include the performance of renal ultrasound and/or MRI^7^. Considering the absence of specific urological symptoms, evaluation of the integrity of the urinary tract is recommended before surgery to plan the best surgical approach and for adequate follow-up after surgery. Pre-operative diagnosis of unilateral kidney failure is important to schedule the surgery and inform the patient of the findings. Moreover, patients with known established unilateral loss of renal function must be followed to ensure there is no ureteral involvement of the contralateral side. Furthermore, it is important to note that all doctors and health caregivers involved in the diagnosis and treatment of endometriosis should be aware of this complication and should suspect and avoid this rare, albeit severe, form of the disease.

Therefore, the aim of this study was to describe DE patients diagnosed with an irreversible unilateral loss of renal function due to ureteral stenosis and to identify risk factors for possibly developing this loss.

## Material and methods

The study was approved by the local Ethical Committee, according to prevailing regulations (Reg. HCB/2020/1152). We conducted a retrospective cohort study including 463 patients who underwent laparoscopic DE surgery due to painful symptoms and/or infertility and/or bowel or ureteral stenosis. We selected all patients who had undergone surgery for DE in our tertiary university teaching hospital between March 2015 and March 2020. We evaluated two groups of patients according to preserved (Non-Renal Loss Group) or irreversibly unilateral irreversible unilaterally damaged renal function (Renal Loss Group). The initial evaluation of DE was performed by transvaginal ultrasound in all cases or magnetic resonance imaging (MRI) when transvaginal ultrasound could not adequately be performed due to pain or incomplete endometriosis stratification information. All patients had histological confirmation of DE. We performed renal and urinary tract evaluation among all patients with endometriosis by sonography or MRI before surgery. When ureterohydronephrosis was clinically suspected (loin pain, dysuria and/or hematuria) or detected on transvaginal ultrasound or MRI, it was evaluated by renal ultrasound. Patients with radiological findings suggestive of hydronephrosis or ureteral stenosis with ureteral dilation of the upstream excretory tract, underwent a renogram^8^. Complete unilateral loss of renal function was defined as relative renal function < 30%^9^.

A descriptive analysis of epidemiologic variables, clinical characteristics, ultrasound findings and surgical treatment was performed. Data were extracted from our institutional electronic medical records.

Before surgery, patients were asked to quantify dysmenorrhea, dyspareunia, dyschezia, dysuria and chronic pelvic pain according to a 0- to 10-point numerical rating scale (NRS) with “0” indicating no pain and “10” indicating the worst possible pain. In order to use the symptoms as potentially predictive we categorized the results into NRS < 7 and NRS ≥ 7 to distinguish between mild/moderate versus severe pain^10^.

The diagnosis of adenomyosis and endometriosis was achieved using 2D-3D transvaginal sonography (TVS), employing an endovaginal probe (type RIC5-9, Voluson V730 Expert, GE Healthcare, Milwaukee, WI, USA). The procedures were performed by two expert sonographers (C.R and M.R) following the Morphological Uterus Sonographic Assessment consensus statement^11^ and the International Deep Endometriosis Analysis consensus^12^.

**Figure 1:**
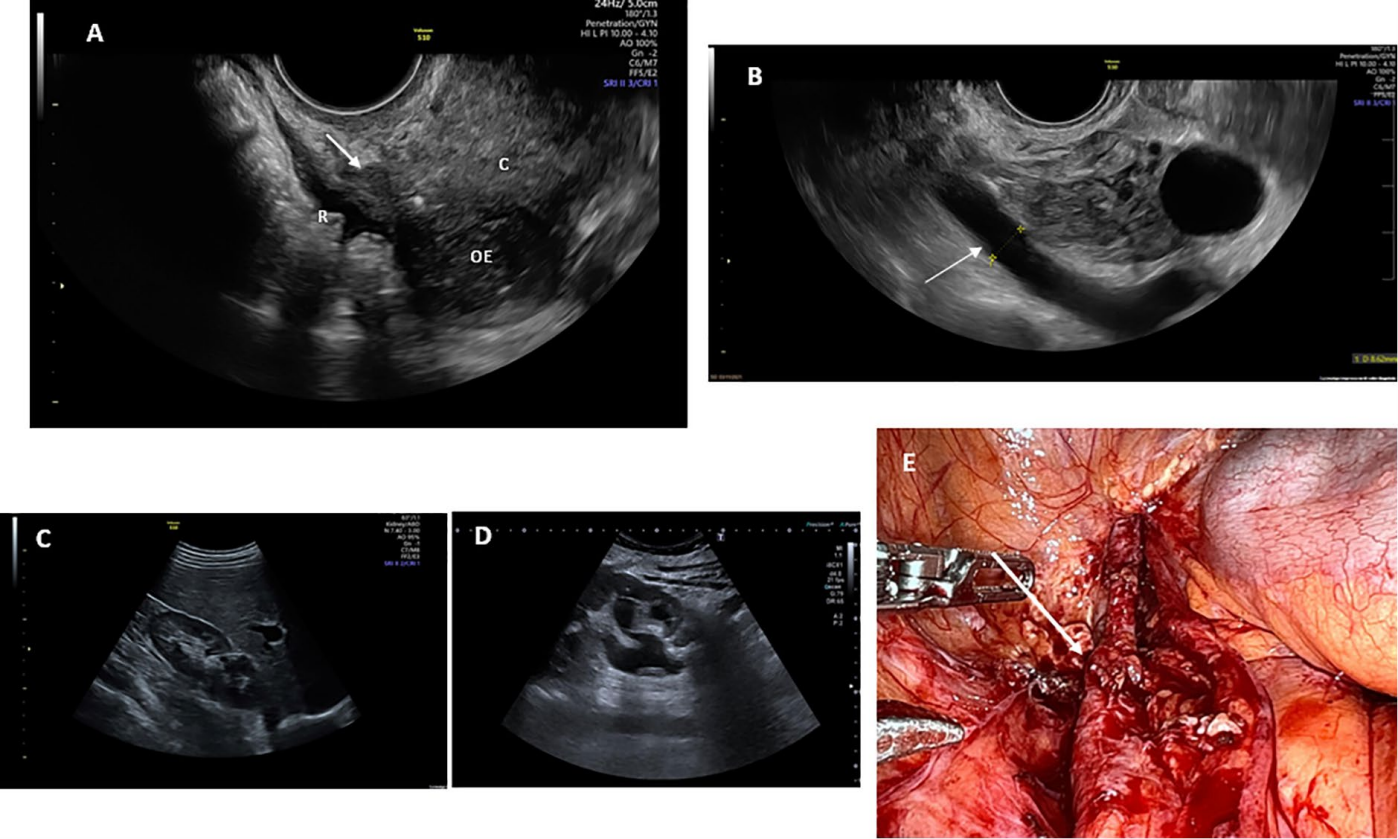
(**A**) Arrow: Sonographic image of hypoechoic thickening of the uterosacral ligament; C: Cervix; R: Rectum; OE: Ovarian Endometrioma. (**B**) Arrow: Sonographic image of ureteral dilatation by the left adnexal space. (**C**) Normal kidney. (**D**) Hydronephrosis secondary to distal ureteral stenosis due to deep endometriosis nodule. (**E**) Surgical image of distal ureteral stenosis (arrow) and upper dilatation due to deep endometriosis pelvic nodule.

To describe the radiologic or sonographic DE findings we used dichotomic and continuous variables: presence versus absence of DE in different locations (i.e., torus uterinus, uterosacral ligaments, bladder, ureter, ovarian endometriomas, etc.), positive or negative sliding sign, and the greatest diameter of DE of each location, and to simplify the total DE burden, the sum of all the largest DE diameters were registered. Bladder DE was considered when patients had endometriosis in the bladder affecting the muscularis. The sliding sign assesses the status of the pouch of Douglas (POD) using real-time TVS. In order to assess the sliding sign when the uterus is anteverted, gentle pressure is placed against the cervix using the transvaginal probe, to establish whether the anterior rectum glides freely across the posterior aspect of the cervix (retrocervical region) and posterior vaginal wall. If the anterior rectal wall does so, the ‘sliding sign’ is considered positive for this location. The examiner then places one hand over the woman's lower anterior abdominal wall in order to ballot the uterus between the palpating hand and the transvaginal probe (which is held in the other hand) to assess whether the anterior bowel glides freely over the posterior aspect of the upper uterus/fundus. If it does, the sliding sign is also considered positive in this region. When the sliding sign is found to be positive in both of these anatomical regions (retrocervix and posterior uterine fundus), the POD is recorded as not being obliterated. If, on TVS, it is demonstrated that either the anterior rectal wall or the anterior sigmoid wall does not glide smoothly over the retrocervix or the posterior uterine fundus, respectively, i.e. at least one of the locations has a negative sliding sign, then the POD is recorded as obliterated. Demonstrating and describing the real-time ultrasound-based sliding sign in a retroverted uterus is different. Gentle pressure is placed against the posterior upper uterine fundus with the transvaginal probe to establish whether the anterior rectum glides freely across the posterior upper uterine fundus. If the anterior rectum does so, the sliding sign is considered to be positive for this location. The examiner then places one hand over the woman's lower anterior abdominal wall in order to ballot the uterus between the palpating hand and transvaginal probe (which is held in the other hand) to assess whether the anterior sigmoid glides freely over the anterior lower uterine segment. If it does, the sliding sign is also considered to be positive in this region. As long as the sliding sign is found to be positive in both of these anatomical regions (i.e. the posterior uterine fundus and the anterior lower uterine segment), the POD is recorded as non-obliterated^12^. Uterosacral ligament DE lesions can be seen in the mid-sagittal view of the uterus. However, these are best seen by placing the transvaginal probe in the posterior vaginal fornix in the midline in the sagittal plane and then sweeping the probe inferolaterally to the cervix (Fig. 1). Uterosacral ligaments are considered to be affected by DE when a hypoechoic thickening with regular or irregular margins is seen within the peritoneal fat surrounding the uterosacral ligaments. The lesion may be isolated or may be part of a larger nodule extending into the vagina or into other surrounding structures^12^.

Statistical analysis was performed with the Statistical Package for Social Sciences software, release 25.0 for Windows (SPSS, Chicago, Illinois). Continuous variables were compared using the nonparametric Mann–Whitney U test or Kruskal–Wallis test using the post hoc Dunn’s multiple comparison test, when appropriate, and presented as median with interquartile range (25th; 75th percentiles). Categorical variables were compared using the Chi-square test and presented as total count and relative percentages (%). Statistical significance was defined as a *p*-value < 0.05. Statistically significant differences among epidemiologic variables, clinical characteristics or radiologic findings were evaluated as independent risk factors using multivariate analysis with logistic regression. Odds ratios (OR) and their 95% confidence intervals (95% CI) were reported.

### Ethical approval

The study was approved by the local Ethical Committee, according to prevailing regulations (Reg. HCB/2020/1152).

## Results

A total of 463 DE patients were identified to be included in the study. Twenty-seven patients did not have accurate information in the clinical records and were excluded. Finally, 436 DIE patients were analyzed. Two groups of patients were evaluated: patients presenting complete unilateral loss of renal function due to endometriosis confirmed by renogram (Renal Loss Group; n = 15) and patients with preserved normal renal function (Non-Renal Loss Group; n = 421). All the patients in the Renal Loss Group had been diagnosed or suspected of renal function loss prior to surgery and most had been referred to our tertiary hospital with the diagnosis of loss of renal function or high suspicion of this complication. The baseline clinical and demographic data of the two study groups are shown in Table 1. There were no differences between the two groups in relation to the mean age, body mass index (BMI), rate of previous surgery (either related or not related to endometriosis), rate of previous hormone treatment, and rate of different pain symptoms. Comparison of clinical data showed that the Renal Loss Group had a higher infertility rate and a higher proportion of asymptomatic patients, being possible variables of suspicion of high risk of loss of renal function due to ureteral stenosis related to endometriosis.

**Table 1:** Baseline clinical and demographic data of the two study groups.

	Non-Renal Loss Group (n = 421)	Renal Loss Group (n = 15)	*p*-value
Age at diagnosis (years)	35.1 (32; 37)	33.8 (28; 38)	0.16
Body mass index (Kg/m^2^)	22.4 (21.1; 24.3)	22.6 (20.3; 25.6)	0.92
Infertility	173 (41.1)	12 (80.0)	0.003
Previous endometriosis surgery	349 (82.8)	10 (66.7)	0.47
Previous non-endometriosis surgery	232 (55.1)	10 (66.7)	0.40
Previous hormonal treatment	240 (57.0)	8 (53.3)	0.77
Asymptomatic	25 (5.9)	4 (26.7)	0.002
Pain symptoms			
Dysmenorrhea (NRS ≥ 7)	280 (66.5)	12 (80.0)	0.29
Chronic pelvic pain (NRS ≥ 7)	117 (27.8)	3 (20.0)	0.49
Dyspareunia (NRS ≥ 7)	109 (25.9)	0 (0.0)	0.06
Dyschezia (NRS ≥ 7)	69 (16.4)	0 (0.0)	0.14
Dysuria (NRS ≥ 7)	19 (4.3)	0 (0.0)	0.46

Results are expressed as median (25th; 75th percentiles) or n (%).

*NRS* numerical rating scale.

Radiologic findings are shown in Table 2. The following variables showed statistically significant differences between the two groups: mean endometrioma diameter, the presence of intestinal DE, negative sliding sign, cumulative DE diameter and bladder DE affecting the muscularis. The presence or absence of adenomyosis or ovarian endometriomas was similar in the two groups. Among the 86 diagnosed ureteral DE, 74 were suspected by imaging before surgery. However, 12 (14%) cases of ureteral DE were diagnosed intraoperatively (Fig. 1). The other ultrasound findings were confirmed during the surgical laparoscopic procedure (Table 2). Nine (2.1%) patients in the Non-Renal Loss Group and 15 (100%) in the Renal Loss Group were diagnosed with hydronephrosis by sonography before surgery (Fig. 1). All these patients underwent renograms.

**Table 2:** Preoperative radiologic data of both study groups.

	Non-renal loss group (n = 421)	Renal loss group (n = 15)	*p*-value
Adenomyosis	226 (53.7)	4 (26.7)	0.06
Ovarian endometrioma	303 (71.9)	7 (46.7)	0.66
Endometrioma diameter (mm)	32.5 (31.1; 54.3)	68.7 (35.2; 87.9)	0.002
Kissing ovaries	65 (15.4)	2 (13.3)	0.84
Intestinal DE	130 (30.9)	9 (60.0)	0.02
Torus Uterinus/Uterosacral ligament DE	176 (41.8)	11 (73.3)	0.01
Bladder DE	11 (2.6)	2 (13.3)	0.02
Ureteral DE	74 (17.6)	15 (100)	< 0.0001
Negative sliding sign	99 (23.5)	10 (66.6)	0.0007
Cumulative DE diameter (mm)	16.6 (12.1; 25.4)	51.1 (21.7; 61.4)	< 0.0001

Values are median (25th; 75th percentiles) or n (%).

*DE* Deep endometriosis.

Table 3 describes the surgical procedures performed in the two groups analyzed. All cases were operated by the same three surgeons (FC, MR and MG). As expected, the surgical procedures in the Renal Loss Group were globally more aggressive, including more adnexectomies, bowel resections and ureteral reimplantations. The histological examination confirmed extrinsic ureteral DE nodules in all patients. No recurrences of ureteral endometriosis were reported during a minimum follow-up of 24 months. Four patients in the Renal Loss Group had bilateral ureteral involvement but unilateral loss of renal function and underwent ureteral reimplantation on the contralateral side.

**Table 3:** Surgical procedures in both study groups.

	Non-renal loss group (n = 421)	Renal loss group (n = 15)	*p*-value
Hysterectomy	46 (10.9)	5 (33.3)	0.05
Uni-Bilateral adnexectomy	54 (12.8)	6 (40.0)	0.02
Uni-Bilateral salpingectomy	69 (16.4)	2 (13.3)	0.50
Bowel resection	36 (8.5)	9 (60.0)	< 0.0001
Endometrioma resection	161 (38.2)	2 (13.3)	0.008
Ureteral reimplantation	16 (3.8)	4 (26.7)	0.002
Nephrectomy	0 (0.0)	5 (33.3)	< 0.0001

Results are expressed as N (%).

In the multivariate analysis, infertility, being asymptomatic, presence of intestinal DE, presence of torus uterinus/uterosacral ligament DE and a negative sliding sign remained significantly associated with irreversible loss of renal function (Table 4).

**Table 4:** Logistic regression analysis of variables associated with complete unilateral loss of renal function in patients with deep endometriosis.

Variable	Adjusted odds ratio	Lower 95% CI	Upper 95% CI	*p* value
Infertility	5.66	1.55	20.69	0.003
Asymptomatic patients	5.95	1.63	21.75	0.014
Intestinal DE	3.69	1.26	10.79	0.018
Torus uterinus/Uterosacral ligament DE	9.89	2.16	45.26	< 0.001
Negative sliding sign	6.55	2.13	20.13	< 0.001

*CI* Confidence interval, *DE* Deep endometriosis.

To identify risk factors for ureteral reimplantation, multivariate analysis was also performed. Patients with torus uterinus/uterosacral ligament DE (odds ratio (OR) 3.7, 95% confidence interval (CI) 1.3–10.1, *p* < 0.04) and a negative sliding sign (OR 5.4, 95% CI 1.5–19.3, *p* > 0.009) were more likely to undergo ureteral reimplantation.

## Discussion

This is the largest report of patients from a single center with unilateral complete loss of renal function due to DE-related ureteral stenosis. We provide variables that may be helpful to preoperatively identify DE patients at high risk of presenting irreversible unilateral loss of renal function based on clinical and radiological findings. Suspicion is indicated by the presence of infertility, being asymptomatic, intestinal DE, torus uterinus/uterosacral ligament DE and a negative sliding sign.

Urinary tract endometriosis may cause ureteral obstruction and lead to complete unilateral renal loss in up to 11.5% of the patients due to its characteristically silent progression, differing from the usual painful symptoms of pelvic endometriosis^3,13,14^. The prevalence of ureteral endometriosis reported in the literature ranges between 0.1 and 1% among all endometriosis patients^15^. Few cases of ureteral endometriosis leading to renal function loss have been described^16–18^. Hence, the prevalence of complete unilateral loss of renal function due to ureteral endometriosis obstruction is, in fact, unknown and is probably underestimated. However, the diagnosis of this involvement has increased over the last years, probably due to higher awareness in specialized endometriosis units and improvement of imaging techniques. Ureteral endometriosis is associated with a risk of subsequent loss of renal function owing to urinary flow obstruction caused by constriction of the caudal portion of the ureter by endometriotic tissue. This flow obstruction is usually unilateral and provokes unilateral loss of renal function. Therefore, accurate and timely diagnosis of ureteral involvement in patients with endometriosis remains a real clinical challenge that requires a high index of suspicion. Considering the absence of specific urological symptoms and the associated risk of silent renal loss, evaluation of the integrity of the urinary tract is recommended in patients with endometriosis, not only before and after surgery but also during medical therapy. There are two major pathological types of ureteral endometriosis: instrinsic and extrinsic, being the latter much more common and found in all the patients in our series. In intrinsic endometriosis, endometriosis tissue infiltrates the muscularis, while extrinsic type endometriotic lesions may be responsible for external ureteral compression. Ureteral stenosis in DE most commonly affects the distal segment of the ureter, i.e. the pelvic ureter at 3–4 cm above the vesico-ureteric junction while less frequently affecting the mid-ureter and, rarely, the proximal ureter^6,16^. This explains why the direct or indirect radiologic/sonographic findings of pelvic DE of the posterior compartment (negative sliding sign, intestinal DE, torus uterinus/DE of the uretosacral ligaments) were found to be high risk factors for unilateral loss of renal function in our study.

Previous studies have attempted to identify risk factors for ureteral obstruction by endometriosis to avoid silent losses in renal function^19–21^, but none have found a specific risk factor to allow early suspicion or a validated preventive diagnostic and therapeutic strategy. Taking into account that there is a high percentage of asymptomatic ureteral involvement in patients with known pelvic endometriosis, it has been suggested that routine urinary ultrasound may ensure early diagnosis of these patients, although cost/benefits should be carefully evaluated^19^. In this sense, previous authors have suggested the possibility of using TVS examination as an accurate non-invasive diagnostic tool for the detection of endometriosis ureteral involvement^22^. Moreover, well-trained gynecologists specialized in endometriosis sonography can perform renal evaluation using transvaginal and/or transabdominal examination to detect/suspect hydronephrosis following well-established recommendations^12^.

Our results are in agreement with the literature regarding the difficulties in diagnosing ureteral endometriosis correctly in the absence of specific symptoms. Conversely, women of reproductive age presenting infertility, pelvic pain and hydronephrosis of unknown cause should be adequately assessed via imaging techniques to achieve high suspicion of ureteral endometriosis^2^. Previous research^13,14,20,23–25^ evaluated endometriosis patients and proposed risk factors for ureteral endometriosis including a lower body mass index^24^, incapacitating dysmenorrhea^25^, parametrial endometriosis^24^, revised American Fertility Society stage IV^20^, rectovaginal DE^13^, retrocervical DE lesions larger than 30 mm^23^, uterosacral ligament endometriosis^14,21^ uterosacral ligament DE lesion ≥ 3 cm in diameter^21^, retrocervical endometriosis and rectosigmoid endometriosis^25^, and previous surgery for endometriosis^20^. Therefore, to sum up and in keeping with previously published results, DE patients mainly with extensive sonographic involvement of the posterior compartment of the pelvis seem to be those at “high risk” and should undergo further renal and ureteral studies. Moreover, it is important to remark that up to 50% of the patients may be asymptomatic^2,5^ and/or infertile^2^. In our series, there were no statistical differences in the percentage of patients under hormonal treatment. Nevertheless, we cannot ascertain the possible protective effect of hormonal treatment to avoid these severe complication because most patients likely started the hormonal treatment when the ureteral obstruction and renal loss were already established.

The main strength of our study was that we compared DE patients with or without complete unilateral loss of renal function with a complete study before laparoscopic surgery. We evaluated a large cohort from a tertiary center to which severe patients are referred, thereby allowing the analysis of a large number of patients with this rare DE complication within a short period of time.

However, this study has several limitations. First, it was based on retrospective data from a single tertiary care center to which severe cases are referred. Therefore, the incidence of loss of renal function among our DE patients was most likely higher than in the general endometriosis/DE population. Second, we evaluated only DE patients, and validation including all types of endometriosis patients should be performed. Finally, to identify high risk patients with loss of renal function it is necessary the use of a precise radiological tool, ideally TVS, to assess different DE locations by a well-trained gynecologist/radiologist, and these tools are usually only available in tertiary referral centers although its use is more and more widespread.

In conclusion, our study showed that being infertile, being asymptomatic, having intestinal DE or torus uterinus/uterosacral ligament DE and a negative sliding sign may help to identify patients with DE at high risk of presenting irreversible unilateral loss of renal function due to ureteral stenosis. Therefore, among these patients, severe urinary tract obstruction should be specifically ruled out with radiological urinary tract tests. Further studies are needed to confirm our results to identify this rare, albeit severe, DE complication and improve surgical planning and endometriosis follow-up.

## Data Availability

All data generated or analysed during this study are included within this published article.
